# Development of 2D Microfluidics Surface with Low-Frequency Electric Fields for Cell Separation Applications

**DOI:** 10.3390/s25185816

**Published:** 2025-09-18

**Authors:** Madushan Wickramasinghe, Dharmakeerthi Nawarathna

**Affiliations:** 1 Department of Electrical & Computer Engineering, Old Dominion University, Norfolk, VA 23508, USA; awick003@odu.edu; 2 Biomedical Engineering Program, Department of Electrical & Computer Engineering, Old Dominion University, Norfolk, VA 23508, USA

**Keywords:** cell separation, sessile droplet, electroosmosis, dielectrophoresis

## Abstract

Cell separation techniques are widely used in many biomedical and clinical applications for the development of screening, diagnosis and therapeutic tests. Current 3D microfluidics-based cell separation methods have limited applications in part due to low throughput and technical complexity. To address these critical needs, we have developed a 2D microfluidics surface which is the miniaturized version of a 3D microfluids cell separation device. Using low-frequency electric fields (1–10 Vpp and 1 kHz–20 MHz), we have first studied dielectrophoresis, AC electro-osmosis and capillary flow within a sessile drop, and finally utilized the results to develop the 2D cell separation surface. Our study has demonstrated that frequency-dependent dielectrophoretic force and AC electro-osmotic flow can be integrated to minimize the capillary flow and subsequently produce clusters of target cells within the 2D microfluidics surface. To demonstrate the concept, we have isolated the blood cells from a red blood cell-lysed blood sample. Cell isolation results show that significant improvement in throughout up to about 120-fold over 3D microfluidics devices. Additionally, due to the technical simplicity, this device offers great potential for use in a wide range of biomedical and clinical applications.

## 1. Introduction

Cell separation is a fundamental technique in biomedical research, diagnostics, bio-sensing and therapy development. The cell separation assays isolate the cell population of interest, i.e., target cells, from cell samples such as blood and tissue [1]. Ideally, cell separation methods require the isolation of target cells with high-throughput, purity and recovery which enable downstream analyses such as single cell omics, e.g., genomics and transcriptomics, high content imaging, flow cytometry, mass cytometry (CyTOF) and digital polymerase chain reaction (PCR) [2,3]. Currently, there is a variety of cell separation methods available to use in assay development. Generally, these cell isolation methods utilize the physical, chemical or biological properties of cells, including size, density, electrical properties and surface markers, to isolate the cell population of interest [4,5]. Density gradient centrifugation is a simpler and more cost-effective method that separates cells based on buoyant density, and it is commonly used for isolating blood cells, but it lacks specificity for the cell type of interest [6]. In contrast, Magnetic Activated Cell Sorting (MACS) employs antibodies coated with magnetic beads to label cells of interest and subsequently isolate cells selectively using magnetic fields. Although this method is fast and scalable, it sometimes produces low purity levels in some applications [7,8]. Fluorescence Activated Cell Sorting (FACS) is a multiparameter method that uses fluorophore markers to label the cells and subsequently identify the labeled cells and isolate them from other cells. FACS is ideal for precise cell sorting, although it is more complex and expensive [9,10]. However, FACS does not have disposable cartridges and therefore it is not recommended for therapeutic applications, where cell samples are infused back to the patient. In addition, other techniques, including adherence-based separation that uses the natural differences in cell adhesion and Laser Capture Microdissection (LCM) that allows precise isolation of cells from tissue sections, have also been developed [11,12]. Recently, microfluidic and label-free concepts such as dielectrophoresis (DEP) [13,14], acoustophoresis [15] and optical trapping [16] have gained attention due to their label-free operation, gentle handling capability of cells, high precision, integrability with downstream analysis and low sample and reagent consumption. Additionally, it has been shown that these methods are ideal for biomedical assay development including medical screening and diagnostics applications. Almost all of these techniques suffer a number of technical issues including low-throughput, purity and recovery, as well as complexity of assays.

In this study, we have developed fundamentally different cell separation methods that could address some of the current technical issues outlined above. Moreover, we studied the feasibility of separating cells suspended in sessile drops. Ideally, this method could eliminate the 3D microfluidic channels, syringe pumps and connection tubing used in current separation techniques and can significantly reduce the technical complexity and subsequently improve the purity, throughput and recovery. Sessile droplets are stationary droplets with volumes up to several milliliters and have internal fluid flow due to capillary flow [17,18]. Capillary flow has been used for developing biomedical assays including analyte concentration [19], cell-based assays [20], particle separation and sorting [21]. Additionally, capillary flow could produce cofree rings (CRs), which can sometimes be disadvantageous for assay development. In addition to capillary flow, we have utilized low-frequency AC electric field (<25 MHz)-driven flow. Ideally, AC or DC electric fields can be used for experiments. However, the magnitude of the DEP force is dependent on the electric polarizability of particles and the electric field magnitude. The electric polarizability is weak in the DC electric fields. Additionally, the magnitude of the electro-osmotic velocity is dependent on the electric field in the buffer. When DC electric fields are used, a significant electric potential will be dropped within the double layer. Therefore, there can be a weak electro-osmotic fluid flow. As a result, we have used AC electric fields in the experiments. The interaction of AC electric fields and biological materials, e.g., cells and biomarker molecules [22,23] and buffers [24], have been extensively studied. AC electric fields polarize the biological materials and buffer in a frequency-dependent manner. The dielectrophoretic force (DEP force) is due to the non-zero electric polarizability of biological materials located in the non-uniform electric fields. DEP force has been extensively used in 3D microfluidics channels to develop cell separation techniques. Depending on the particle’s dielectric properties and the AC frequency, particles exhibit either positive (movement toward high ∇(E^2^)) or negative DEP (movement away from high ∇(E^2^)), which enables frequency-selective discrimination between micro-particles, e.g., cancerous vs. healthy cells or live vs. dead cells [25,26]. Additionally, electro-osmotic flow within the sessile drop is due to the charging of the double layer by the AC electric field [27]. The capillary flow is generally toward the edge of the sessile drop and produces CR at the edge [28]. It has been demonstrated that when an AC voltage is applied across asymmetric microelectrodes, it creates a steady electro-osmotic flow that can generate vortices or directional fluid movement to enhance transport and pre-concentration of particles [24]. In this study, we have studied whether the interactions of AC electric fields with cells and buffer, combined with a capillary flow, can be used to develop cell separation. Moreover, we have investigated whether frequency-dependent DEP force and AC electro-osmotic drag force on the cells can be integrated with the capillary flow to separate the particles. Below, we discuss the separation assays, calculations and experiments developed by integrating DEP force, AC electro-osmotic drag force and capillary flow to separate polystyrene beads and blood cells.

## 2. Materials and Methods

### 2.1. Comsol Calculations

AC electro-osmotic flow calculation was performed using COMSOL Multiphysics software 5.1 (Burlington, MA, USA). First, T-electrodes and sessile drop (accommodate about 50 µL of cell sample) were drawn to scale and an electric current module (AC/DC) was used to calculate the electric potential distribution within the drop followed by electric fields. We then used the Creeping flow interface to calculate the flow velocity within the droplet.

### 2.2. CM Factor Calculation

Real parts of the Clausius and Mossotti factor (CM) at each frequency of interest were calculated using the formula listed below [29].

(1) $$Re\left[CM\left(\omega \right)\right]=\frac{\omega^{2}\left({\varepsilon_{0}\varepsilon }_{p}-\varepsilon_{0}\varepsilon_{m}\right)\left(\varepsilon_{0}\varepsilon_{p}+2{\varepsilon_{0}\varepsilon }_{m}\right)+(\sigma_{p}-\sigma_{m})(\sigma_{p}+2\sigma_{m})}{\omega^{2}{\left(\varepsilon_{0}\varepsilon_{p}+2\varepsilon_{0}\varepsilon_{m}\right)}^{2}+{\left(\sigma_{p}+2\sigma_{m}\right)}^{2}}$$

where subscripts *m* and *p* represent medium and particle, respectively, *ω* is the angular frequency of the applied electric field, *ε* is the permittivity, *σ* is the conductivity and *r* is the particle radius. The following values were used in the calculations: particle permittivity $(\varepsilon_{p})=2.6,\;medium\;permitivity\;(\varepsilon_{m})=78$; medium conductivity $(\sigma_{m})=\;20\;\mu S\;{cm}^{-1}$. For the polystyrene beads, the particle conductivity ${\;(\sigma }_{p})$ values were calculated using

(2) $$\sigma_{p}=\sigma_{bulk}+\frac{2K_{s}}{r}$$

We have neglected the conductivity of the medium ($\sigma_{bulk})$; the surface conductance of the particle is ${(K}_{s})=2\times 10^{-9}\;S$ [30]. Similarly, for blood cells, we have used the published values, e.g., for while blood cells $\varepsilon_{p}=133\;$ and $\sigma_{p}=0.6\;S\;m^{-1}$ [31].

### 2.3. Polystyrene Bead Separation Experiments

#### 2.3.1. Sample Preparation

Commercially available 1 µm, 3 µm, 5 µm and 10 µm diameter polystyrene beads (Phosphorex LLC, Hopkinton, MA, USA and Polysciences, Warrington, PA, USA) were used in bead separation experiments. The working bead samples were prepared by diluting the original bead solutions in a buffer and washing the beads by centrifugation to remove ions and surfactants in the original bead solution. Moreover, bead samples were suspended in diluted Tris EDTA (TE) buffer (Thermo Fisher Scientific, Ward Hill, MA, USA) with the conductivity of 20 µs/cm (0.002×). Additionally, tween 20 (Fisher bioreagents, Somerville, NJ, USA) was added to produce tween 0.002% in the bead sample by volume. Working solutions with bead concentrations of 1070 beads/µL were prepared for each bead size and used for the preparation of the final bead solutions.

Bead samples were prepared by mixing working solutions of different bead sizes by maintaining ~5% of 10 µm beads over the total amount of beads. Additionally, the total bead amount was kept approximately constant (~53,000 beads per 50 µL droplet or 1.06 × 10^6^ beads/mL) in all the experiments.

Binary bead sample (1 µm and 10 µm): 25 µL of 10 µm beads mixed with 475 µL of 1 µm beads.

Tertiary bead sample (1 µm, 5 µm and 10 µm): 25 µL of 10 µm beads were mixed with 237.5 µL of 5 µm beads and 237.5 µL of 1 µm beads.

Quaternary bead sample: (1 µm, 3 µm, 5 µm and 10 µm): 25 µL of 10 µm beads were mixed with 158 µL of 5 µm beads, 158 µL of 5 µm beads and 158 µL of 1 µm beads.

For the enrichment vs. bead concentration experiments, binary mixtures of 5 µm and 10 µm diameter beads were prepared by changing the total bead concentration in each experiment and by maintaining 0.5% of the 10 µm beads ratio.

#### 2.3.2. Polystyrene Bead Separation Experimental Parameters and Instrumentation

A total of 50 µL of a bead sample was pipetted onto the T-electrode array and the following electric field sequence was applied immediately.

- 1 Vpp 125 kHz for 5 s;
- 9 Vpp 20 MHz for 20 s;
- 9 Vpp 1 MHz for 5 s;
- 5 Vpp 1 kHz for 1 s.

A signal generator (Tektronix AFG-1062, Beaverton, OR, USA) automated with in-house NI LabVIEW program (2020 Spring, Auston, TX, USA) was used to apply the sequence of electric fields. When the electric field sequence was applied, two clusters of beads started to form on either side of the droplet and 2.5 µL of each cluster (total of 5 µL) was extracted at the 15th cycle using a pipette, diluted to 150 µL with DI water, and 50 µL of the diluted sample was analyzed using the Flow Cytometer (MACSQuant10, Bergisch Gladbach, Germany). Control samples were prepared by extracting 5 µL volume from the original sample and diluting to 150 µL with DI water, and a similar analysis procedure to that discussed above in the flow cytometer was followed.

### 2.4. Cell Separation Experiments

#### 2.4.1. Cell Sample Preparation

Blood samples were purchased from Innovative Research Inc. (Peary Court, Novi, MI, USA), and the Abcam 10X Red Blood Cells (RBC) lysis buffer (Abcam, Waltham, MA, USA) was used to lyse the red blood cells. To visualize the cells during the separation, cells were stained using a commercially available live and dead cell imaging kit (Life Technologies Corporation, Eugene, OR, USA).

RBC were lysed by following the Abcam 10X RBC lysis buffer protocol provided by the manufacturer. For the experiments with lower cell concentrations, first, 20 mL of 1X RBC lysis buffer was added to 1 mL of human whole blood, left for incubation at room temperature for 5 min and then centrifuged (Thermo Electron LED GmbH, 37520 Osterode am Harz, Germany) at 500× *g* for 5 min at room temperature. Then, the supernatant was removed and resuspended the pellet in 500 µL of diluted PBS. Similarly, for the experiments with higher cell concentrations, two tubes were prepared with each tube having 1 mL of whole blood and 10 mL of 1X RBC lysis buffer and each pallet was resuspended in 250 µL of diluted PBS (conductivity = 20 μS/cm), and they were mixed to form 500 µL final sample volume. The cell sample was stained with live dead cell imaging kit and used the fluorescence microscope to visualize the cell sample during separation. Cell staining was performed using manufacturer-provided assay.

#### 2.4.2. Cell Separation Experimental Parameters and Instrumentation

In total, 50 µL of cell sample was pipetted onto the T-electrode array, and the following electric field sequence was applied immediately.

- 3 Vpp 10 kHz for 5 s;
- 1 Vpp 1 kHz for 5 s;
- 9 Vpp 1 MHz for 10 s;
- 9 Vpp 100 kHz for 5 s.

The signal generator (Tektronix AFG-1062, Beaverton, OR, USA) automated with the in-house NI LabVIEW program (2020 Spring, Auston, TX, USA) was used to apply the sequence of electric fields. Over time, a single cluster of cells started to form in the droplet and 5 µL from the cell cluster was extracted at the 15th cycle using a pipette, diluted to 150 µL with 1× PBS, and 50 µL of the sample was analyzed using the Flow Cytometer (MACSQuant10, Bergisch Gladbach, Germany). An analysis of the sample before applying the electric field was performed using 5 µL volume from the cell sample and adding 145 µL of diluted PBS buffer and analyzed the sample in the flow cytometer.

### 2.5. Beads and Cell Sample Analysis

#### 2.5.1. Flow Cytometry

The flow cytometry was performed using MACSQuantify (Miltenyi Biotec, Bergisch, Germany, version 2.13.3) by setting up gates to produce Forward scattering (FSC) vs. Side Scattering (SSC) plots. The particles in each sample were used to calculate the enrichment and recovery values.

#### 2.5.2. Enrichment Calculation

We calculated % cells in the P3 region before and after the separation. We then calculated the enrichment in folds using (percentage cells in the P3 region after the separation/percentage cells in the P3 before separation).

#### 2.5.3. Recovery Calculation

First, the number cells in the region P3 in the pre-separate sample (per 50 µL droplet) was calculated from the flow cytometer data (N*_Control_*). Second and finally, after the separation experiments, the total extracted in cells in the P3 population was calculated (N*_Extracted_*). Recovery was calculated as a % using (N*_Extracted_*/N*_Control_*) × 100.

## 3. Results

**Figure 1:**
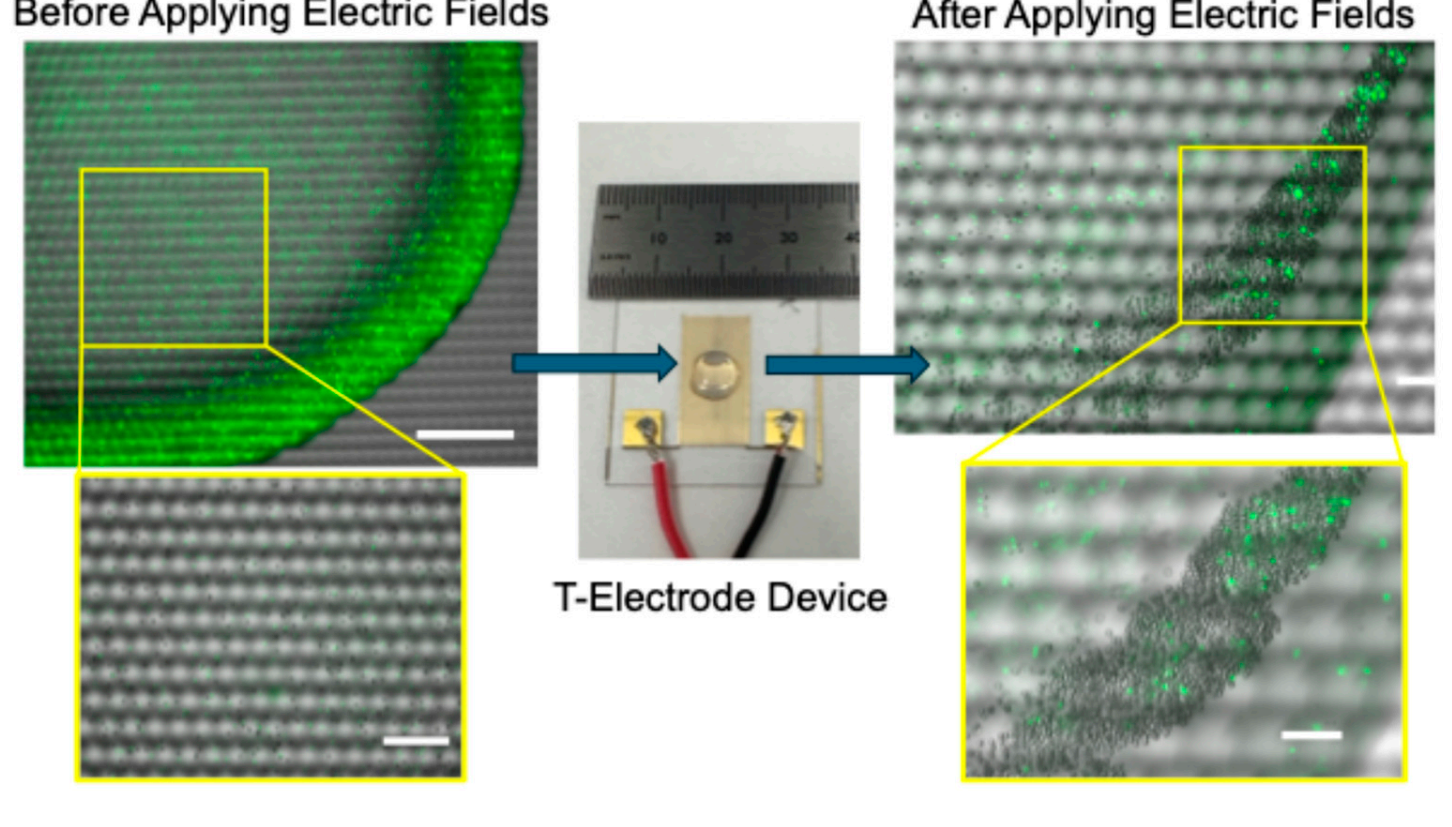
Schematic representation of the experiment set up used in the study. Briefly, first, the cell or bead sample was pipetted onto the device that had an interdigitated T-electrode array. Second, a sequence of AC electric fields was applied. Note that each electric field was applied for a pre-determined time interval (1–10 s); the electric field sequence was repeated for about 15 cycles and the cell or bead cluster formation was observed. Finally, a clustered cell or bead sample was carefully extracted from the device using pipetting and quantified using flow cytometry. Images show the separation of 10 μm diameter polystyrene beads from a binary mixture of 5 μm (fluorophore-labeled; shown in green color) and 10 μm diameter polystyrene beads. Scale bars: The left side of the image shows 250 μm and the right side of the image shows 100 μm.

Figure 1 illustrates the experimental set up and the device used in the experiments. Interdigitated T-electrodes were designed and manufactured by our group for biomedical engineering applications [32,33]. The T-electrodes were made of gold using traditional photolithography methods. Details of the electrode fabrication were discussed elsewhere [34]. Earlier, we demonstrated that T-shape is critical for producing large electric fields (E) and the electric field gradients ∇(E^2^) needed for strong DEP forces on cells and beads [34]. To demonstrate the concept, we have conducted experiments using a binary mixture of 10 μm and fluorophore-labeled 5 μm diameter polystyrene beads suspended in a diluted (conductivity: 20 µs/cm) TE buffer. We have used a 1:1 bead ratio in experiments. As demonstrated in Figure 1, prior to separation, a cell or bead sample was carefully pipetted onto the middle of the electrode array, and subsequently, the electric field sequence discussed in the Materials and Methods section was applied. During the experiments, the device with the sample was placed on a microscope stage (Keyence BZ-X810, Itasca, IL, USA) for continuous monitoring of the particle motion, cluster formation and completion of the separation. We have found that cluster formation was needed to achieve enrichment as well as to extract the sample from the electrodes. Figure 1 shows (left of the device) an image of a bead sample before the electric field sequence was applied. Note that there are a large number of fluorophore-labeled polystyrene beads present in the sample. Once the electric field sequence was applied and waited for cluster formation, the image shows (right of the device) the bead cluster. Note that in comparison, a smaller number of fluorophore-labeled beads were collected in the bead cluster. A large number of non-fluorescence polystyrene beads were collected in the cluster. As noted, enrichment of non-fluorescence polystyrene beads was achieved. The negative control experiments were performed by pipetting the bead sample on the electrodes and leaving the sample on the electrodes without applying the electric fields. No separation or cluster formation was achieved, but CRs were formed due the capillary flow.

When a single electric field was applied, depending on the frequency of the electric field, making bead patterns throughout the electrode array or bead cluster formation was possible but the enrichment of target beads was not good. Therefore, initial experiments were carried out using a sequence of electric fields. Moreover, a sequence of electric fields with steps for DEP force-based concentration of beads, flushing out unwanted beads from the electrodes and finally producing a cluster was needed. The experiments conducted with a sequence of electric fields also did not yield a good enrichment value. We then repeated the sequence of the electric fields, which created continuous separation conditions similar to the particle separation in 3D microfluidics channels. Moreover, in the electric field sequence, we applied the DEP force, fluid flow or viscous drag forces on the particles in series manner, and in 3D microfluidics, those forces are applied in a parallel manner. We have found that the frequency of each electric field step was critical to the success of the separation. For example, for the DEP force-based concentration of target beads on the electrodes, it is required to know the frequency at which the DEP force is maximum for the beads of interest (called the target). At the same time, it is also required to know about DEP forces on the other particles (called non-targets) in the sample. Ideally, when the DEP force of the target particle is maximum, the DEP forces of the other particles should be minimum. The time-averaged DEP force of a spherical bead or cell that has a of *r* can be written as

**Figure 2:**
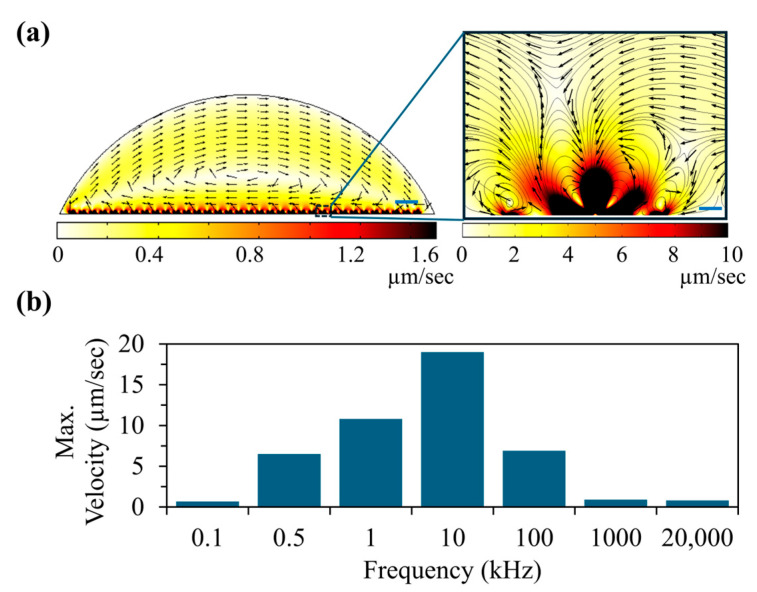
The calculation of fluid velocities produced by AC electro-osmosis (ACEO) in a 50 μL sessile drop (conductivity: 20 μS/cm) pipetted on the interdigitated T-electrode array. The calculations were performed using the COMSOL software (version 5.1). (**a**) ACEO flow pattern in the droplet when a sinusoidal signal (1 kHz, 5Vpp) was applied to electrodes. The space plot illustrates the magnitude of the fluid velocity and normalized arrows show the direction of the fluid flow (scale bar 400 μm). The inset shows the zoomed view of the ACEO flow near a single electrode pair. (**b**) Variation in the maximum ACEO flow velocity with the frequency of the applied electric field (scale bar 20 μm). Note: 1 Vpp sinusoidal signal was used in the calculation.

FDEP=2πr3εmRe(CM)∇ERMS2

where CM is the Clausius and Mossotti factor, *E* is the electric field and *ε_m_* is the dielectric permittivity of the medium. The DEP force can be positive (pDEP) or negative (nDEP) which is based on the sign of the Re(CM). Note that if the sign of Re(CM) is negative, nDEP is expected and vice versa. We have found that for polystyrene beads, nDEP force is expected in the low frequency range (<20 MHz). The other important frequency is the flushing frequency needed to remove the trapped unwanted beads from the electrodes. To flush out the unwanted beads from the electrodes, we have used the AC electro-osmotic drag force on particles, which was a result of AC electro-osmosis. Studies have shown that AC electro-osmotic drag force can be used to manipulate particles in sessile drops as well as in microfluidics channels [35,36]. The magnitude of the drag force is dependent on the frequency and tangential electric field produced by the electrodes. Moreover, electro-osmotic fluid flow velocity is given by the following formula, $\frac{E_{t}\sigma_{p}}{\kappa \eta }$, where *E_t_* is the tangential electric field, *σ_p_* is the surface charge in the diffuse layer, *κ* is the reciprocal Debye length and *η* is the viscosity of the buffer [37]. To further understand the AC electro-osmosis flow within the droplet, we have calculated the fluid flow velocities in a range of frequencies that we expect to use in experiments (Figure 2). We have found that strong fluid velocities can be expected in the low frequency range (<100 kHz). Figure 2a shows the calculated fluid flow distribution within the sessile drop at 1 kHz. The inset shows the close-up view near the electrodes. Based on this calculation, at 1 KHz, average fluid flow velocities up to 25 μm/s can be expected near T-electrodes. This means that the high viscous drag force on the polystyrene beads near the electrode can provide better cleaning up. Additionally, this calculation shows that AC electro-osmotic flow velocity diminishes in the areas that are away from the electrodes. To further understand the variation in fluid flow velocity with the frequency of the applied electric field, we extended our calculation to other frequencies (see Figure 2b). To understand the frequency-dependent fluid flow calculations, the characteristic frequency (rad/s) of the buffer was calculated using *σ*/*ε* where *σ* and *ε* are the conductivity and dielectric permittivity of the buffer, respectively, and the calculated characteristic frequency was ~460 kHz. We have found that AC electro-osmotic fluid velocity was higher in the frequencies closer to the characteristic frequency, which is consistent with the other published studies [27,37]. In addition to the frequency, the magnitude of the electric field and the time applied are also important for particle separation. For example, note that during the flush step, both target and non-target beads can flush away, but the target beads can be kept on the electrodes by reducing the flush time and increasing the DEP force of the target beads.

**Figure 3:**
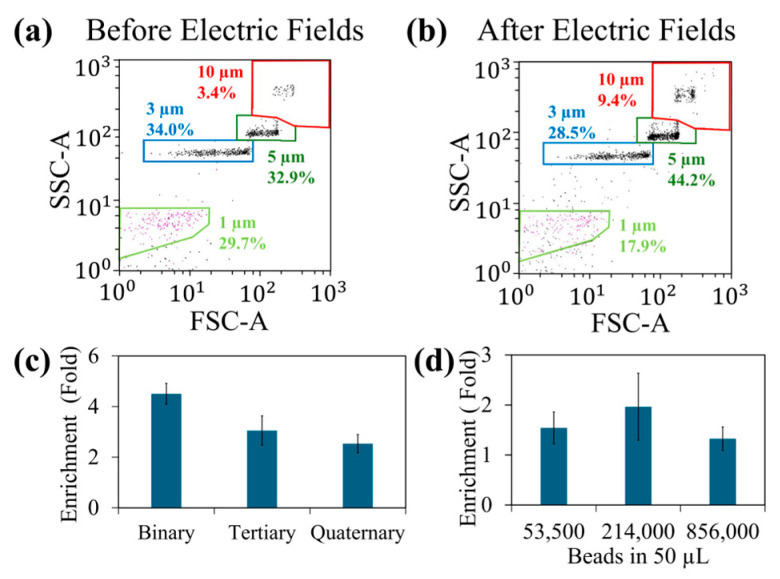
Demonstration of polystyrene bead separation using a sequence of electric fields. Figure (**a**,**b**) show the effects of electric fields on polystyrene bead sample. (**a**) Composition of the bead sample (50 μL with 53,500 beads) with no electric field sequence. This analysis was performed using FSC vs. SSC feature of flow cytometry. (**b**) The composition of the beads from the sample collected after applying the sequence of electric fields for about 7.5 min. Note that this bead sample was collected from the beads cluster formed after applying the electric fields. (**c**) The experimental enrichment values of 10 μm polystyrene beads in binary (10 μm and 1 μm), tertiary (10 μm, 5 μm and 1 μm) and quaternary (10 μm, 5 μm, 3 μm and 1 μm) bead samples. Each sample had about 5% of 10 μm beads in 53,500 total beads (in 50 μL) and exposed to the sequence of electric fields about 7.5 min. The bead sample was collected from the bead cluster formed after applying the electric fields. (**d**) Variation in enrichment of 10 μm polystyrene beads with the total number of polystyrene beads. Each experiment was performed using a binary mixture of 10 μm and 5 μm beads and each sample had about 0.5% of 10 μm beads. The bead sample was exposed to the sequence of the electric fields about 7.5 min, collected the clustered bead sample and quantified the sample using flow cytometry (FSC vs. SSC).

Next, based on the AC electro-osmotic drag force and DEP force on polystyrene beads, we have designed a sequence of electric fields to be used in experiments. To demonstrate the concept, we have used a binary mixture of 1 μm (non-target) and 10 μm (target) diameter beads in experiments. The selection of bead sizes was based on the DEP force of the beads. Moreover, at a given frequency, the DEP force on 10 μm beads should be 1000 times bigger than that of 1 μm beads. At the same time at a given fluid flow velocity, the viscous drag force of 10 μm beads is 10 times bigger than that of 1 μm beads. To concentrate beads on the T-electrodes using DEP force, based on our CM factor calculation, MHz frequencies can be used. We have selected 20 MHz of the electric field that can concentrate beads on the electrode as the CM factors of 1 μm and 10 μm at this frequency are −0.47 and −0.47, respectively. Note that at this frequency, AC electro-osmotic fluid flow is <2.5 μm/s. The issue with this frequency is the collection of non-target beads, i.e., 1 μm beads. To reduce the accumulation of 1 μm beads, we added another electric field step that has a frequency of 1 MHz. CM factors of 1 μm and 10 μm at 1 MHz are 0.15 and −0.42, respectively. Furthermore, at this frequency 10 μm beads can have ~3000 times bigger DEP force than the 1 μm beads. Additionally, at this frequency, the AC electro-osmotic fluid flow is <2.5 μm/s. We expect less trapping of 1 μm beads compared to 10 μm beads. Next, to flush out non-target beads, based on our AC electro-osmotic fluid flow calculation, we have used 1 kHz frequency to produce fluid flow as our calculation shows maximum fluid velocity of 10 μm/s. Finally, the bead cluster formation is a result of both AC electro-osmosis and DEP force. Therefore, low-to-intermediate frequency can be used to produce clusters and therefore ~125 kHz can be used. We have used these frequencies in the initial experiments and magnitude of the electric field and time of each electric field step was adjected to optimize the enrichment of 10 μm beads. The optimized electric field sequence was then used to separate 10 μm beads from binary (10 μm and 1 μm), tertiary (10 μm, 5 μm and 1 μm) and quaternary (1 μm, 3 μm, 5 μm and 10 μm) bead mixtures. Figure 3 illustrates the summary of the findings.

Figure 3a,b show the quantification of bead samples before and after exposure to the electric field sequence. We have determined the values for the magnitude of each electric field and the on-time of each electric field experimentally to achieve the best enrichment values. Note that 10 μm beads were enriched by the application of the electric field sequence discussed above.

Additionally, 5 μm beads also had a small enrichment. This could be due to the trapping of 5 μm beads with 10 μm beads as their DEP force ratio at 20 MHz is 1:8. In contrast, 1 mm and 3 μm beads quantities have decreased in the exposed samples. Figure 3c shows the variation in enrichment with various bead samples. The binary mixture had the greatest enrichment as the significant DEP force difference between 1 μm and 10 μm beads. The enrichment values were decreased when other bead samples that had bead sizes closer to the size of 10 μm beads. Next, we studied the influence of number of beads per sample in the enrichment. When the number of beads were high, it can saturate the T-electrodes and minimize the bead trapping. Additionally, beads crowding within the droplet can also reduce the trapping efficiency. On the other hand, if the bead sample was too diluted, the beads trapping can be less efficient. This is because DEP force is only efficient in capturing beads located near the electrodes. Figure 3d shows the variation in enrichment with bead concentration. Ideally, to achieve the maximum enrichment, it is required to design electrode density to concentrate all the beads or cells of interest. Additionally, if the enrichment is poor, that sample can be pipetted back to the electrodes and re-run the separation. We have also found that the electric field sequence does not have to be applied in a certain order. Rather, due to the repeating of the sequence, it can apply any order that we want.

In the next set of experiments, we studied whether this concept can be extended to separate cells from a real-world sample, i.e., blood sample. The selection of cells in the cluster is based on their DEP force. Moreover, the cell type that has largest DEP force will be concentrated in the cluster. The previously used electric field sequence was modified to accommodate the DEP forces of the blood cells. To modify the electric field step related to DEP force, we have recalculated CM factors of blood cells using the published dielectric properties of the blood cells and they were ~1 in the frequencies used in this experiment. We have selected 1 MHz and 100 kHz to produce strong DEP force on blood cells as in these frequencies AC electro-osmotic flow velocities are small. Additionally, to produce AC electro-osmotic fluid flow, we have used 1 kHz as used in previous bead separation experiments. To produce cell clusters, the frequency that produces both DEP force and the AC electro-osmotic flow is needed, and we have used 10 kHz frequency. As discussed earlier, the magnitude and application time of each electric field were also important for the success of the cell separation. We have initiated experiments using the parameters that we used in the bead separation and further optimized to maximize the enrichment. The optimized protocols used for separation of blood samples were discussed in the Materials and Methods section.

**Figure 4:**
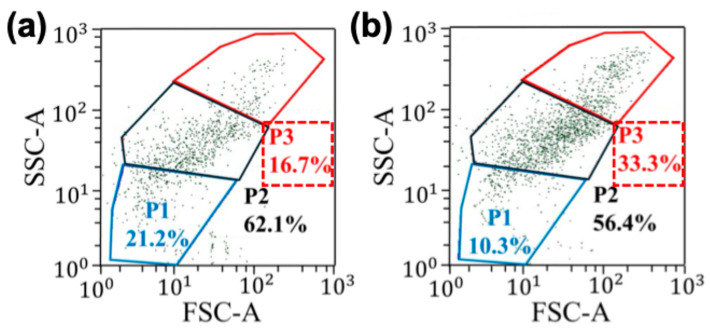
Effects of the RBC-lysed blood sample after exposure to the sequence of electric fields produced by interdigitated T-electrodes. The cell sample was exposed to the electric fields for about 6 min, and the cell cluster was extracted by pipetting and analyzed using flow cytometry (FSC vs. SSC). (**a**) Quantification of the cell sample pipetted on the electrodes before applying the electric field sequence. (**b**) Quantification of cell cluster extracted from the cell sample after exposing the sequence of electric fields for about 6 min. Note that population P3 was enriched by the application of electric fields while other populations (P1 and P2) were negatively impacted by the sequence of electric fields.

**Figure 5:**
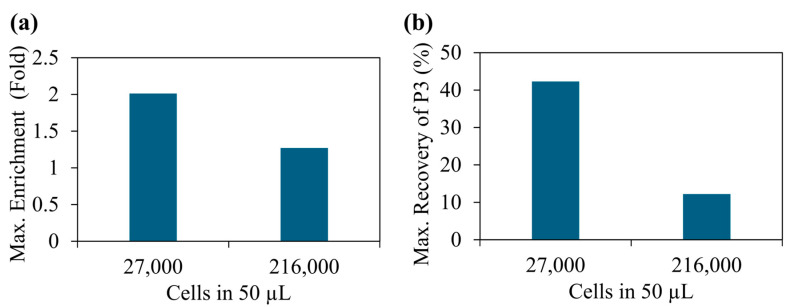
Variation in enrichment and recovery of the P3 population with the total number of cells. Each sample was exposed to the sequence of electric field for about 6 min, and the cluster was extracted and analyzed using flow cytometry (FSC vs. SSC). (**a**) Variation in maximum enrichment of population P3 with cell concentrations. (**b**) Variation in maximum cell recovery of population P3 with cell concentrations.

Figure 4 shows the summary of the separation experiments performed using the electric field sequence discussed above. Figure 4a and b show the quantification of a cell sample before and after applying the electric field sequence, respectively. Note that application of electric field sequence influenced the enrichment of the cell population in area P3. Additionally, P1 represents lymphocytes, P2 represents monocytes and P3 represents granulocytes [38]. In comparison, cell populations in areas P1 and P2 were reduced. Based on the FSC vs. SSC plot, the cell population in the region P3 had the largest-sized cells. This data shows that DEP force was maximum for the largest cell size. The throughput of the cell separation is about 300 µL/h (or 50 µL per 10 min). In contrast, studies that used traditional DEP and magnetophoretic cell separation techniques to isolate blood cells have produced the throughput values of 50 µL/h and 2.5–20 µL/h [39,40]. Therefore, a maximum improvement in the throughput of about 120-fold (~300/2.5) can be achieved with the 2D microfluidics surface. Next, we studied the variation in cell enrichment with the number of cells in the sample (Figure 5a). Figure 5a illustrates the maximum values, but the average values were 1.83 (±0.13) 1.10 (±0.15) for 27,000 and 216,000 beads, respectively. These values were calculated independently, repeating each experiment at least three times. We have observed a significant variation in enrichment with the number of cells in the sample. When we had about 27,000 cells in 50 mL (540,000 cells/mL), a maximum of two-fold enrichment was achieved. In contrast, when the cell concentration was increased to about 216,000 (5.4 × 106 cells/mL), no significant enrichment was produced. Previously, as discussed in the bead separation experiments, the variation in the cell enrichment could be due to particle crowding near electrodes and saturation of DEP force-based particle trapping. Additionally, this data suggests our previous claim about optimizing cell concentration to control the cell density of the electrodes. To further improve the enrichment, multi-step separation can be used. Briefly, one can collect the sample from the first stage of separation and pipette it back to the electrode and separate again. In each step, the enrichment could increase. Furthermore, enrichment can also be improved by fine-tuning the electric field sequence used in the experiments. Another important factor to study is cell recovery, which is the number of target cells (e.g., cell population in region P3) recovered after the separation. We conducted additional experiments and studied the recovery of our separation method. Figure 5b shows the variation in target cell recovery vs. cell concentration. The average values of the recovery were 40% (±2%) and 11% (±1%) for 27,000 and 216,000 beads, respectively. We have repeated each experiment at least three times. When the cell concentration was optimized to effectively accommodate electrodes, a maximum recovery of ~42% was recorded. In contrast, the traditional Ficoll separation method that used blood samples similar to our samples produced about 50–70% recovery values [41]. To further improve the recovery, the electric field sequence needs to be further optimized. In particular, steps related to DEP force trapping and cluster formation could further improve the recovery. These results highlight the need for developing a sample-specific electric field sequence to achieve high enrichment and recovery.

## 4. Conclusions

In this study, we have demonstrated that dielectrophoresis and AC electro-osmosis can be integrated to cause cell separation. Additionally, the capillary flow from center to the edge of the sessile drop was minimized using the integration discussed above. Compared to traditional methods such as centrifugation or magnetic separation, due to the simplicity of the technique, this sessile droplet-based separation called 2D cell separation surface can be attractive to point-of-care applications. The integration of forces discussed above resulted in an efficient cell and bead separation in sessile drops with sample volumes up to 50 µL. However, the separation technology can be scaled up to analyze other sample volumes beyond 50 µL. To scale up the separation, the area of electrodes needs to be increased. We have used interdigitated T-electrodes in experiments to apply electric fields and subsequently produce the electric field gradients needed for separation. However, these experiments can also be performed with other types of electrodes. Additionally, further optimization of T-electrode density, i.e., number of T-electrodes per µm^2^, will also help to improve the enrichment and recovery values. The primary parameter that differentiated the target cells from others was the DEP force of the cells. Moreover, to separate specific particles (larger polystyrene microbeads or cells), the relevant dielectric properties of the particle of interest should be known and included in the DEP force trapping. Dielectric properties of the commonly used cells (e.g., cancer cells) and molecules (e.g., DNA molecules) have been identified and reported by others [42,43,44]. Therefore, future cell separation studies using our technology can benefit from the published studies. Furthermore, other cellular parameters can also be used to isolate cells on the electrodes, e.g., electric polarizability at a certain frequency or cell type-specific frequency to produce DEP forces specifically on the cell type of interest. Additionally, this separation method is not limited to DEP force. For example, magnetophoretic force can also be integrated with AC electro-osmosis and capillary flow to separate magnetically labeled particles.

Compared to traditional methods such as centrifugation or magnetic separation, due to the simplicity of the technique, this sessile droplet-based cell separation can be attractive to many biomedical applications including point-of-care applications. However, there are some technical challenges to be addressed before integrating with clinical settings. First, the device needed to be optimized to be compatible with larger sample volumes and this can be performed by increasing the size of the electrode array. Second, performance needs to be further investigated, especially when the cell density is higher. Once cell separation protocols are optimized, automation of the assay will also need to be developed. Finally, clinical samples must be analyzed and the results must be verified.

## Data Availability

The original contributions presented in this study are included in the article. Further inquiries can be directed to the corresponding author.
